# DrwH, a novel WHy domain-containing hydrophobic LEA5C protein from *Deinococcus radiodurans*, protects enzymatic activity under oxidative stress

**DOI:** 10.1038/s41598-017-09541-2

**Published:** 2017-08-24

**Authors:** Shijie Jiang, Jin Wang, Xiaoli Liu, Yingying Liu, Cui Guo, Liwen Zhang, Jiahui Han, Xiaoli Wu, Dong Xue, Ahmed E. Gomaa, Shuai Feng, Heng Zhang, Yun Chen, Shuzhen Ping, Ming Chen, Wei Zhang, Liang Li, Zhengfu Zhou, Kaijing Zuo, Xufeng Li, Yi Yang, Min Lin

**Affiliations:** 1 Key Lab of Bio-resources and Eco-environment of Ministry of Education, College of Life Sciences, Sichuan University, Chengdu, PR China; 2 Biotechnology Research Institute, Chinese Academy of Agricultural Sciences, Beijing, China; 3 Plant Biotechnology Research Center, School of Agriculture and Biology, Shanghai Jiao Tong University, Shanghai, China

## Abstract

Water stress and hypersensitive response (WHy) domain is typically found as a component of atypical late embryogenesis abundant (LEA) proteins closely associated with resistance to multiple stresses in numerous organisms. Several putative LEA proteins have been identified in *Deinococcus* bacteria; however their precise function remains unclear. This work reports the characterization of a *Deinococcus*-specific gene encoding a novel WHy domain-containing hydrophobic LEA5C protein (named DrwH) in *D*. *radiodurans* R1. The expression of the *drwH* gene was induced by oxidative and salinity stresses. Inactivation of this gene resulted in increased sensitivity to oxidative and salinity stresses as well as reduced activities of antioxidant enzymes. The WHy domain of the DrwH protein differs structurally from that of a previously studied bacterial LEA5C protein, dWHy1, identified as a gene product from an Antarctic desert soil metagenome library. Further analysis indicated that in *E*. *coli*, the function of DrwH is related to oxidative stress tolerance, whereas dWHy1 is associated with freezing-thawing stress tolerance. Under oxidative stress induced by H_2_O_2_, DrwH protected the enzymatic activities of malate dehydrogenase (MDH) and lactate dehydrogenase (LDH). These findings provide new insight into the evolutionary and survival strategies of *Deinococcus* bacteria under extreme environmental conditions.

## Introduction

Bacteria of the genus *Deinococcus* are among the most radiation-resistant organisms found on Earth. In particular, the *Deinococcus radiodurans* strain R1 was the first identified deinobacteria exhibiting remarkable resistance to a variety of stresses, including gamma radiation, UV light, desiccation, heat, and oxidation^1–3^. Resistance to ionizing radiation observed in *Deinococcus* may results from its ability to resist to extreme desiccation^2^. It has been postulated that these bacteria possessed specialized genetic systems for DNA repair and response to stresses to account for their extreme resistance^3–5^. Other hypotheses proposed that the extreme resistance of *Deinococcus* was resulting from horizontal transfer of genes. The analysis of the *D*. *radiodurans* genome revealed the existence of at least 15 genes likely transferred horizontally from unexpected sources, such as eukaryotes and viruses^3^. Moreover, there is increasing evidence that genes coding for the proteins involved in the radiation-desiccation resistance properties of *Deinococcus* bacteria might originate from other extremophile bacteria after horizontal transfer^6–10^. It is particularly noteworthy that three *Deinococcus* genes (designated *dr1372*, *dr0105* and *dr1172*) encode homologs of plant desiccation-related late embryogenesis abundant (LEA) proteins^3, 7^.

LEA proteins, originally found in plants, are a group of heterogeneous proteins accumulating under various stress conditions like drought, salinity, extreme temperature and oxidative stress^11–17^. Subsequently several genes encoding putative LEA proteins have been found in bacteria^18, 19^, such as *Bacillus subtilis* ^20^ and *D*. *radiodurans* ^3, 21^. The LEA protein families have diverse structures and functions. Generally, typical LEA proteins are characterized by repeat motifs, structural disorder and high hydrophilicity in their native forms^17, 22, 23^. In contrast to the typical LEA proteins, the LEA proteins from the group 5 display relatively high hydrophobicity but lack significant signature motifs and are thus considered atypical LEA protein^24, 25^. In particular, the LEA5C proteins, found in plants, contain a **W**ater stress and **Hy**persensitive response (WHy) domain, suggesting that they are probably functionally different from typical LEA proteins^16, 26, 27^. There is only one report on the functional characterization of a bacterial protein containing a WHy domain, dWHy1^28^. The structural gene coding for this protein was identified from an Antarctic desert soil metagenome library and it was found that dWHy1 displayed *in vivo* protection against cold and freeze damage^28^.

*D*. *radiodurans* has been extensively used as a model bacterium to study the resistance to irradiation and desiccation, but the underlying mechanisms of its extreme resistance are still poorly understood. Several putative LEA proteins have been found in *Deinococcus* bacteria, but their role has not been experimentally investigated. We report here the characterization of the *drwH* gene, a *Deinococcus*-specific LEA5C gene from *D*. *radiodurans* R1. The *drwH* gene encodes a novel hydrophobic protein, named DrwH, containing a novel WHy domain. Expression of the *drwH* gene was significantly up-regulated in response to oxidative and salinity stresses and its expression was under control of the global regulator IrrE via unknown mechanisms. The *drwH* mutant displayed increased sensitivity to oxidative and salinity stresses as well as reduced activities of antioxidant enzymes. Secondary structure prediction and enzyme protection assays of the WHy domain from the *D*. *radiodurans* DrwH and the bacterial dWHy1 from an Antarctic desert soil metagenome library revealed the evolutionary and functional diversity of the LEA gene family in bacteria under extreme environmental conditions. These results indicated that DrwH protects enzymatic activity from damage caused by oxidative stress, probably contributing to the extreme tolerances of *D*. *radiodurans*.

## Results

### DrwH is a novel hydrophobic LEA5C protein containing a WHy domain

**Figure 1:**
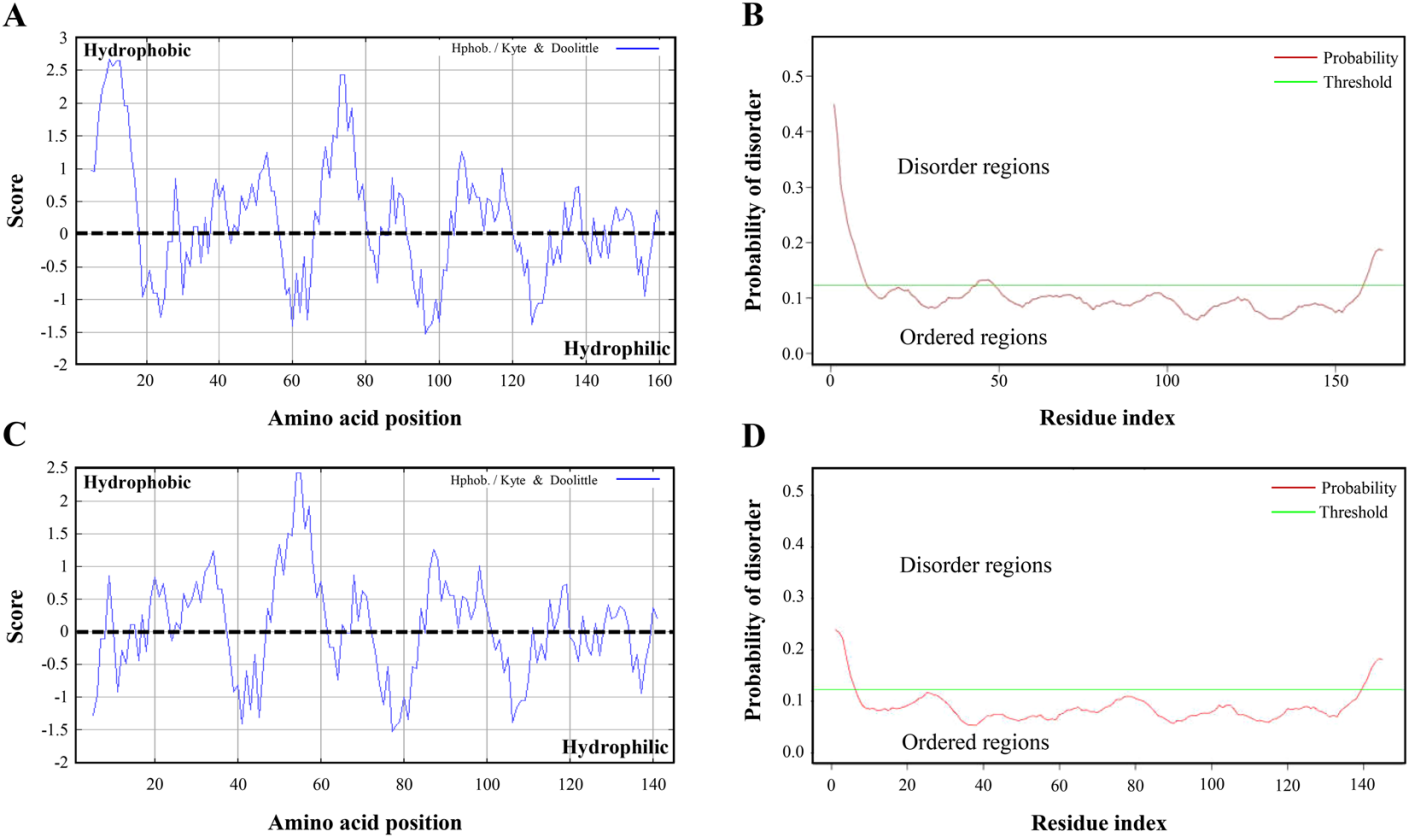
Hydropathy and disorder prediction of intact and truncated DrwH proteins without putative signal peptide. (**A**) Hydropathic index plot of the deduced DrwH amino acid sequence analyzed using the Kyte–Doolittle algorithm. Regions with a hydropathy score above zero are hydrophobic. (**B**) Prediction of DrwH disordered regions using Cspritz. Regions with scores above the threshold line are considered to be disordered. (**C**) Hydropathic index plot of the deduced amino acid sequence of truncated DrwH analyzed using the Kyte–Doolittle algorithm. (**D**) Prediction of truncated DrwH disordered regions using Cspritz.

Analysis of the *D*. *radiodurans* genome revealed the presence of a 495-bp open reading frame (ORF), *dr1372*, coding for a putative LEA protein. Phylogenetic analysis indicated that the DR1372 protein and its homologs only found in *Deinococcus* genomes are clustered into a small subgroup distinct from that of plants and archaea (see Supplementary Fig. S1). DR1372 is rich in hydrophobic residues, such as Leu (13.4%), Ala (11.6%), Val (11.0%), and Pro (9.1%), while lacking Cys and Trp residues. As shown by hydropathy plots^29^, the grand average hydropathy (GRAVY) value for DR1372 was predicted to be 0.220, suggesting a relatively high level of overall hydrophobicity (Fig. 1A and Supplementary Table S1). Furthermore, bioinformatics analysis revealed the presence of a signal peptide located at the N-terminal end and a WHy domain with an invariant triplet “NPN” motif from the 36^th^ to 136^th^ residues (see Supplementary Fig. S2). The DR1372 protein disorder predictions suggested a small propensity to be an order protein using Cspritz software (Fig. 1B). The predicted hydropathy and ordered characteristics of the truncated DR1372 protein without the signal peptide showed a very high degree of similarity to the intact DR1372 protein (Fig. 1C,D). These results, together with the phylogenetic tree of DR1372 and related protein sequences (see Supplementary Fig. S1), indicate that DR1372 belongs to the LEA5C group. The other LEA5C characterized protein of bacterial origin, dWHy1^28^, also containing a WHy domain, cluster in the *Pseudomonas* genus subgroup (see Supplementary Fig. S1). A total of 27 *Deinococcus* proteins had a relatively high similarity (57.2–79.3%) with DR1372. No DR1372 homolog was found in any other bacterial species. In addition, DR1372 had very low similarity (<30%) with the LEA proteins previously identified (see Supplementary Figs S1 and S2). Because we did not identify homologs in any other bacterial species by a BLAST analysis and did not find any match to the LEA families in the Pfam database, DR1372 is likely a novel *Deinococcus*-specific LEA5C protein containing a WHy domain. We therefore renamed this protein DrwH.

### The *drwH* gene is significantly induced by oxidative and salinity stresses

**Figure 2:**
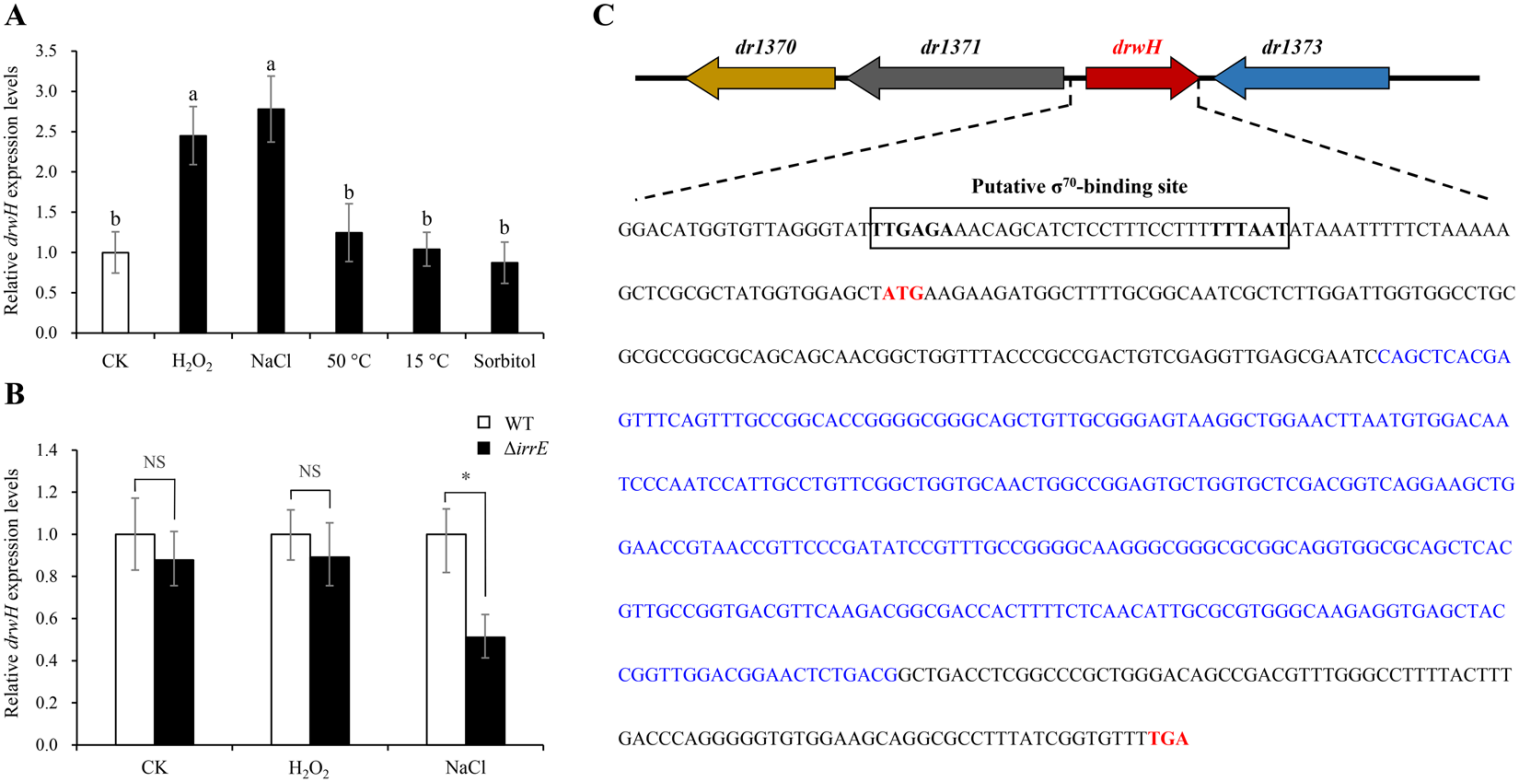
Transcriptional analysis of *drwH* in *D*. *radiodurans*. (**A**) Relative expression level of *drwH* in response to various stresses in *D*. *radiodurans*. Total RNA of *D*. *radiodurans* was extracted after exposure to 50 mM H_2_O_2_ (30 min), 0.3 M NaCl (2 h), 50 °C (2 h), 15 °C (2 h), and 0.5 M D-sorbitol (2 h). Different letters indicate significant differences (*P* < 0.05). (**B**) *drwH* transcription in *D*. *radiodurans* WT and Δ*irrE* mutant strains under oxidative and salt conditions. Error bars represent the standard error of the mean of 3 independent experiments. The asterisk indicates a significant difference, which was calculated with Student’s t-test (*P < 0.05, NS: no significant difference). (**C**) Nucleotide sequence of the putative promoter region of *drwH*. The open boxes represent the putative σ^70^-dependent promoter. The start and termination codons of the *drwH* gene are indicated in red bold letters. The 300-bp region containing a predicted WHy domain is highlighted in blue.

Bacterial LEA proteins are thought to be involved in generalized stress responses. To test the function of the *drwH* gene, the expression pattern of the *drwH* gene was examined after exposure to various stresses similar to those encountered under extreme environments for *Deinococcus*. Cultures of *D*. *radiodurans* R1 were subjected to oxidative stress (induced by H_2_O_2_), osmotic stress (induced by NaCl and D-sorbitol), heat and cold shocks. Total RNA was isolated and the *drwH* expression was detected by quantitative real-time PCR (qRT-PCR) analysis. As shown in Fig. 2A, *drwH* expression was significantly up-regulated in response to oxidative and salinity stresses. After 30 min treatment with 50 mM H_2_O_2_, *drwH* expression was induced by nearly 2.5-fold. In addition, a 2.8-fold increase in response to salt stress was observed. No significant expression changes were detected after treatment with 0.5 M D-sorbitol and different temperature treatments. *D*. *radiodurans* genome encodes only one predicted σ^70^ factor (RpoD/SigA, DR0916), which thus plays a major role in transcription of growth-related genes^30^. Sequence analysis of the *drwH* promoter region revealed a presence of a putative typical −35/−10 consensus sequence (TTGAGA-N20-TTTAAT) (Fig. 2C), suggesting that it may be a target site recognized by the σ^70^ factor. Previous studies have shown that IrrE is a global regulator involved in salt tolerance^31, 32^. To determine whether *drwH* is regulated by IrrE, we checked the expression levels of *drwH* in the *D*. *radiodurans* Δ*irrE* mutant strain previously constructed^33^. As shown in Fig. 2B, the expression of *drwH* was down-regulated by 2-fold in the Δ*irrE* mutant background under salt stress, whereas no significant difference was observed under oxidative stress. These results suggest that the expression of the *drwH* gene is significantly induced by oxidative and salinity stresses and under the control of IrrE via unknown mechanisms during salinity stress.

### Deletion of *drwH* decreases abiotic stress tolerances of *D. radiodurans*

**Figure 3:**
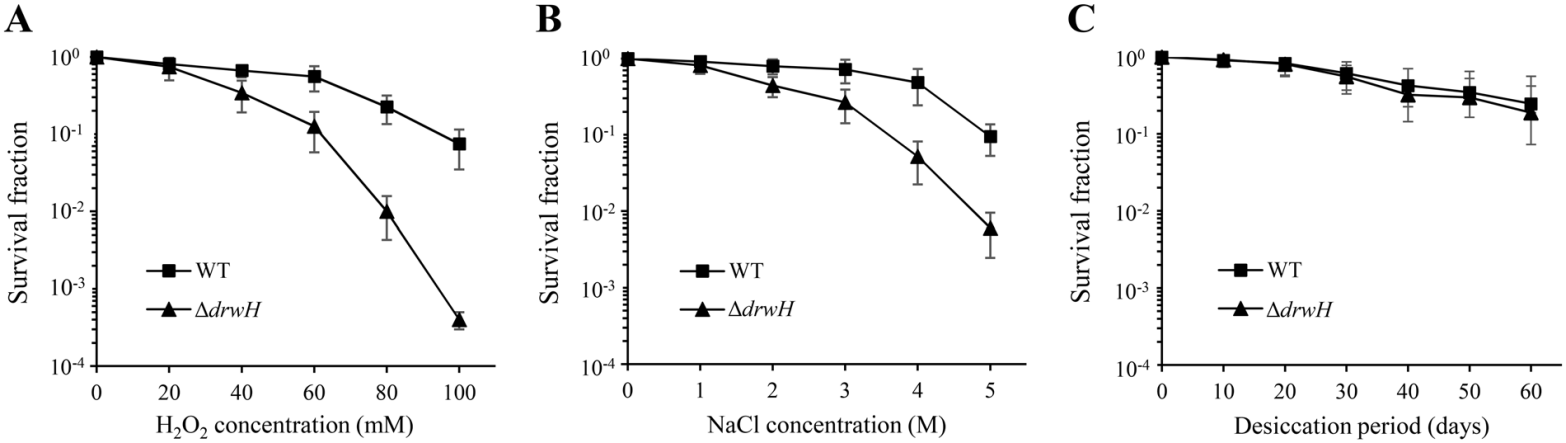
Survival curves for *D*. *radiodurans* WT and Δ*drwH* mutant following exposure to H_2_O_2_ (**A**), NaCl (**B**), and desiccation (**C**) treatments. Different dilutions of these cells were plated on TGY agar plates and incubated at 30 °C for 3 days before colonies were enumerated. The survival rate was expressed as the percentage of the number of colonies in the treated samples compared with those in untreated controls. All experiments were performed three times and are represented as mean ± standard deviation.

The *drwH* gene, which was found to be significantly up-regulated in response to abiotic stresses (Fig. 2), is most likely involved in the extreme stress tolerance phenotype of *D*. *radiodurans*. To assay this hypothesis, a *drwH* deletion mutant strain (Δ*drwH*) was constructed using a kanamycin-resistance-gene (*nptII*) replacement strategy (see Supplementary Fig. S3)^34^. To examine whether the *drwH* gene deletion has an effect on the expression of the flanking genes (*dr1371* and *dr1373*), we compared the expression levels of *dr1371* and *dr1373* in *D*. *radiodurans* WT and Δ*drwH* mutant by qRT-PCR. We found that expression of *dr1371* and *dr1373* was up-regulated by ~5 fold and 2 fold in the Δ*drwH* mutant, respectively. This observation suggests that the deletion somehow affected the flanking gene expression, possibly due to the global effect of the *drwH* gene deletion. The phenotype of the mutant strain is due to the lack of the DrwH protein and consequent changes in the transcription of affected genes, including the upregulation of the expression of *dr1371* and *dr1373*. Then we performed stress tolerance assays as indicated in Fig. 3. The survival growth curves showed that the Δ*drwH* mutant strain was more susceptible to increasing H_2_O_2_ concentrations than *D*. *radiodurans* wild-type (WT) strain (Fig. 3A). Furthermore, the absence of *drwH* caused a significant decrease in the survival rates of the Δ*drwH* mutant strain when the cells were treated with NaCl (ranged from 0 to 5 M). The rate of survival, 5 hours after a 4 M NaCl shock treatment, was only of 5.2% for the Δ*drwH* mutant strain while it was of 48.5% for *D*. *radiodurans* WT under the same treatment (Fig. 3B). TGY plate assays for oxidative and salinity stresses led to similar findings as shown in Supplementary Fig. S4. In general, LEA proteins accumulate during late embryogenesis in seeds, but it is also expressed in vegetative tissues and its expression can be induced by various abiotic stresses such as desiccation, salinity, extreme temperatures and oxidative stress^18, 22, 23, 25^. Unexpectedly, the Δ*drwH* mutant response to desiccation at 5% humidity of up to 60 days was similar to that of WT (Fig. 3C). These results suggested that the *in vivo* function of DrwH is related to both oxidative and salinity stress tolerances, but not to desiccation stress.

### DrwH is required for the expression and activity of antioxidant enzymes

**Figure 4:**
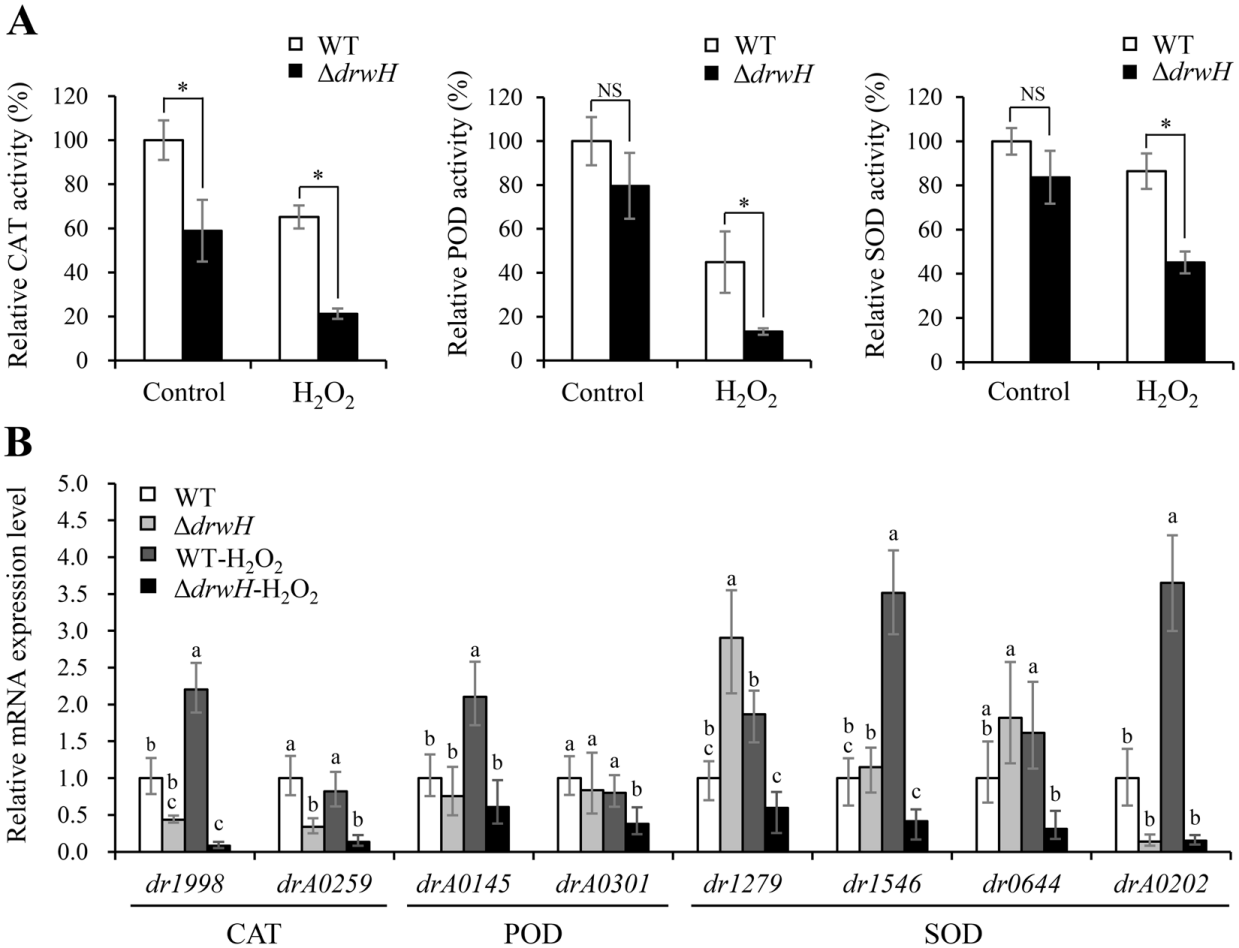
Expression and catalytic activities of ROS scavenging enzymes in *D*. *radiodruans* WT and Δ*drwH* mutant strains under oxidative stress. (**A**) Effects of the *drwH* deletion on the enzyme activities after treatment with 50 mM H_2_O_2_ for 30 min. The asterisk indicates a significant difference, which was calculated with Student’s t-test (*P < 0.05, NS: no significant difference). (**B**) Effects of *drwH* deletion on the expression of antioxidant enzyme genes after treatment with 50 mM H_2_O_2_ for 30 min. All experiments were performed three times and are represented as mean ± standard deviation. Different letters indicate significant differences (*P* < 0.05).

Various detoxifying enzymes, which serve to protect cells from the toxic effects of reactive oxygen species (ROS) generated by oxidative stress, were proposed to contribute to the *D*. *radiodurans* extreme resistance phenotype^35^. We thus assessed the effect of *drwH* deletion on the expression and activity of ROS scavenging enzymes, such as catalase (CAT), peroxidase (POD) and superoxide dismutase (SOD). Consistent with their oxidative sensitive phenotypes, the Δ*drwH* mutant strain showed lower expression and activities of the three ROS scavenging enzymes than did the WT strain after treatment with 50 mM H_2_O_2_ for 30 min (Fig. 4A). The CAT, POD and SOD activities showed decreases of 3-, 3.4-, and 1.8-fold, respectively. In addition, two genes (*dr1998* and *drA0259*) encoding CAT, two genes (*drA0145* and *drA0301*) encoding POD, and four genes (*dr1279*, *dr1546*, *dr0644* and *drA0202*) encoding SOD were significantly down-regulated in the Δ*drwH* mutant strain under oxidative stress, consistent with the decreased activities of the antioxidant enzymes (Fig. 4B). Together, these results suggested that DrwH is required for expression and activity of ROS scavenging enzymes and likely contributes to the extreme tolerances of *D*. *radiodurans*.

### Structural and functional analysis of the WHy domain

**Figure 5:**
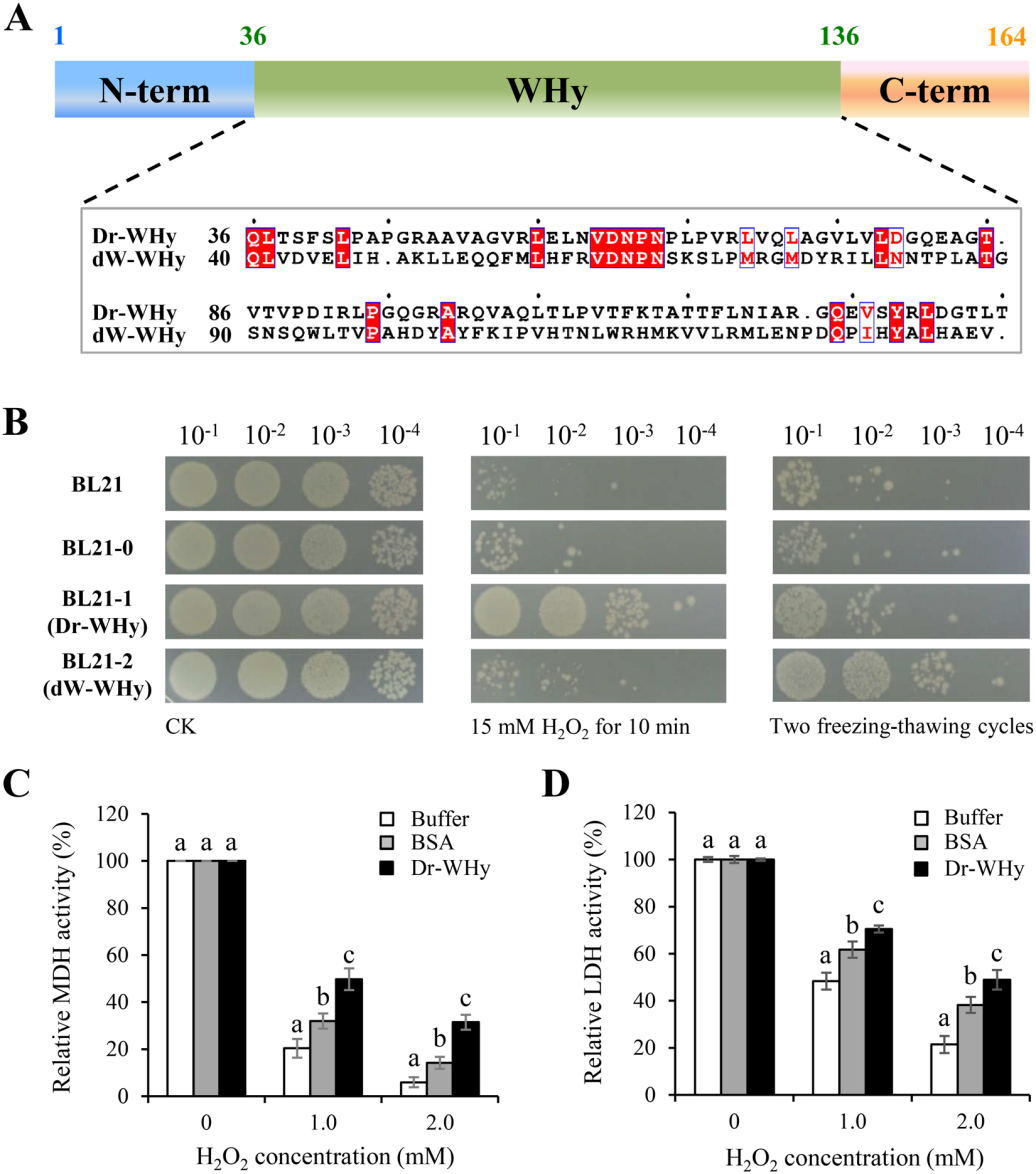
Structural and functional analysis of the WHy-domains from DrwH and dWHy1. (**A**) Primary structure analysis of the DrwH protein, and the alignment between the WHy-domains from DrwH and dWHy1. (**B**) Survival phenotype plate assay of the *E*. *coli* wild-type strain BL21 and recombinant strains expressing Dr-WHy (truncated DrwH, BL21-1), dW-WHy (truncated dWHy1, BL21-2), and an empty vector (BL21-0) as a control, respectively, after treatment with 15 mM H_2_O_2_ for 10 min and two freezing-thawing cycles. (**C**) and (**D**) Protective effect of Dr-WHy on MDH and LDH activities after H_2_O_2_ treatment. The His-tagged Dr-WHy (truncated-DrwH) protein was used for the *in vitro* protection assays. Each column represents an average of three independent experiments, and error bars represent standard deviation. The statistical difference was tested by Student’s t-test (*P* < 0.05). Different letters indicate significant differences.

To date, only two bacterial water hypersensitivity-like proteins, DrwH from *D*. *radiodurans* and dWHy1 from an Antarctic desert soil metagenome library, have been experimentally characterized. The sequence similarity of the WHy domains from DrwH and dWHy1 was compared using ClustalX2 software, and they shared only 33.3% sequence similarity (Fig. 5A). Furthermore, the secondary structure prediction indicated that the WHy domain of DrwH structurally differs from that of dWHy1 (Table 1). Since the WHy domain from DrwH showed low sequence similarity to dWHy1, we hypothesized that the DrwH protein may have a different function from dWHy1. Bioinformatics analysis suggested the presence of a predicted signal peptide in both DrwH and dWHy1. Anderson *et al*. (2015) have previously shown that the survival rates were significantly higher under stress conditions for the *E*. *coli* expressing the complete wild-type dWHy1 protein, and even higher when the truncated dWHy1 protein without the predicted signal peptide was expressed^28^. This is consistent with our observation that expression of the complete wild-type DrwH protein significantly decreased growth of recombinant *E*. *coli*, regardless of the presence or absence of H_2_O_2_ (see Supplementary Fig. S5). Since expression of wild-type protein with the predicted signal peptide affected *E*. *coli* growth, we constructed the truncated protein with an intact WHy domain but without the predicted signal peptide and performed *in vivo* stress tolerance assays or *in vitro* enzyme protection assays.

**Table 1:** Secondary structure prediction for the WHy domains of the *D*. *radiodurans* DrwH and the bacterial dWHy1 from an Antarctic desert soil metagenome library

WHy domain	Alpha helix (%)	Extended strand (%)	Beta turn (%)	Random coil (%)
Dr-WHy	15.00	38.00	5.00	42.00
dW-WHy	36.00	23.00	7.00	34.00

To test this, we constructed a truncated-DrwH (Dr-WHy) strain BL21-1 and a truncated-dWHy1 (dW-WHy) strain BL21-2, with intact WHy domains but without the predicted signal peptides, and examined their growth under abiotic stresses. After freezing-thawing stress treatment, BL21-2 (dW-WHy) displayed significantly enhanced tolerance to freezing-thawing stress, whereas *E*. *coli* wild-type BL21 and BL21-1 (Dr-WHy) strains did not (Fig. 5B). This result is consistent with the previous observation that expression of dWHy1 in *E*. *coli* significantly protected the recombinant host against cold and freezing stresses^28^. Following treatment with 15 mM H_2_O_2_ exposure for 10 min, overexpression of Dr-WHy significantly enhanced oxidative resistance of *E*. *coli*, whereas dW-WHy had no effect (Fig. 5B). These results suggest that the *in vivo* function of DrwH is related to oxidative stress tolerance, whereas dWHy1 is associated with freezing-thawing stress tolerance. Collectively, secondary structure prediction and *in vivo* stress tolerance assays of the WHy domains from DrwH and dWHy1 revealed the evolutionary and functional diversity of the bacterial LEA family under different extreme environments.

To further evaluate the potential protective effect of the WHy domain, we carried out *in vitro* assays to test the effects of Dr-WHy on the enzymatic activities of malate dehydrogenase (MDH) and lactate dehydrogenase (LDH) following exposure to oxidative stress induced by H_2_O_2_. Bovine serum albumin (BSA) and potassium phosphate buffer were used as positive and negative controls, respectively. As shown in Fig. 5C, MDH retained only 10% residual activity following exposure to 1.0 or 2.0 mM H_2_O_2_. However, the addition of Dr-WHy efficiently prevented inactivation, with the samples retaining 30–50% of their initial activity. Similar protective effects of Dr-WHy were also observed when LDH was subjected to oxidative treatment (Fig. 5D). BSA is a well-known cryoprotectant protein, which also had protective effects on MDH and LDH but it was much less efficient than Dr-WHy. The *in vitro* results suggested that the intact WHy domain efficiently protects MDH and LDH activities from inactivation caused by oxidative stress.

## Discussion

Until now, the complete genome sequencing has been established for 23 *Deinococcus* spp., including *D*. *radiodurans* R1 isolated from canned meat spoiled following exposure to X-rays^36^, *D*. *geothermalis* DSM11300 from a hot spring^8^, *D*. *actinosclerus* BM2 from soil of a rocky hillside^37^, *D*. *maricopensis* LB-34 from a soil sample from the Sonoran Desert in Arizona^38^, *D*. *deserti* VCD115 from the Sahara surface sands, an extreme and nutrient-poor environment^39^, *D*. *peraridilitoris* KR-200 from a sample of arid soil collected from a coastal desert in Chile^40^, and *D*. *gobiensis* I-0 from the cold Gobi desert^9^. These species can survive when exposed to extreme conditions, such as acute irradiations, cycles of extreme temperatures, extreme desiccation and numerous oxidizing agents, known to cause protein damage lethal to most organisms on Earth. Therefore, *Deinococcus* bacteria should possess an extraordinary adaptive strategy to withstand environmental extremes. Comparative genomic and transcriptome analyses indicated that *Deinococcus* bacteria contain a surprisingly large number of horizontally acquired genes, some of which are induced by various abiotic stresses, suggesting that their extreme resistance phenotype may be attributable to still unknown genes and pathways^6, 8–10^. In this study, we functionally characterized a novel bacterial WHy domain-containing hydrophobic LEA5C protein, DrwH, which is induced by oxidative and salinity stresses. Since DrwH is specific to the genus *Deinococcus* and can protect various intracellular enzymes as shown by *in vivo* stress tolerance assays or *in vitro* enzyme protection assays (Figs 4 and 5), it is implicated in the extreme tolerance of *Deinococcus* bacteria. Based on the results presented here and previous literature reports, we proposed a working model for DrwH in *D*. *radiodurans*, which tentatively illustrates its ability to effectively protect activities of various intracellular enzymes from damage caused by oxidative stress (see Supplementary Fig. S7).

The DrwH protein is a novel *Deinococcus*-specific LEA5C protein with a relatively high level of overall hydrophobicity, a certain degree of cytotoxicity to the growth of *E*. *coli* and *D*. *radiodurans*, and a pleiotropic role in *D*. *radiodurans* physiology. These characteristics have generated confusion and difficulties in studying the function of the *drwH* gene, particularly in complementation experiments. We constructed a complementation strain using the wild-type *drwH* gene with its endogenous promoter and performed complementation assays under oxidative stress (see Supplementary Fig. S8). We observed that the complementation strain grew almost similar to the wild type and mutant strains under normal growth conditions. However, plasmid-mediated complementation resulted in an obviously decreased sensitivity to hydrogen peroxide, and the complementation strain was significantly more sensitive than the wild type and mutant strains (see Supplementary Fig. S8B). On the other hand, we compare expression level of the *drwH* gene between the wild type and complementation strains. Unexpectedly, although the *drwH* gene possesses its endogenous promoter in the complementation strain, its transcriptional level is much higher than that of *D*. *radiodurans* WT following exposure to 80 mM H_2_O_2_. As observed in Supplementary Fig. S5, expression of the wild-type DrwH protein significantly decreased growth of recombinant *E*. *coli*, suggesting the cytotoxicity of the DrwH protein to the cell growth. We thus suspect that the overexpression of *drwH* in the plasmid-mediated complementation strain could result in reduced growth under oxidative stress, but it requires further investigation.

Generally, the function of LEA family proteins can be explained by two molecular mechanisms, namely the chaperone-like action^41^ and the molecular shield activity^42^. In some instances, LEA proteins function as a chaperone-like molecular shield reducing aggregation of target proteins, suggesting that both mechanisms may not be mutually exclusive^41^. The protective capacity of the typical hydrophilic LEA proteins is due to their ability to bind to interaction partners accompanied by a folding transition, such as disorder-to-a mostly α-helix conformation upon drying^43, 44^. Similarly, chaperone-like activities have been reported for MtPM25, an atypical LEA protein that dissociates cold and desiccation-aggregated proteins^24^. This chaperone-like activity could be explained by the hydrophobic nature of MtPM25 together with the high water sorption capacity of the disordered conformation, which both facilitate or induce transient hydrophobic interactions with exposed surfaces of the aggregates, as suggested by Haaning *et al*.^45^. To date, no precise function has been attributed to the *drwH* gene. On the basis of the protective action of the hydrophobic LEA proteins as discussed above, DrwH might stabilize proteins by a chaperone-like mechanism involving transient hydrophobic interactions; however, this possibility has not yet been examined experimentally.

The WHy domain is widespread among plants, and also detectable in some bacteria and a few euryarchaeota species^26, 27^. This sporadic occurrence can be explained by horizontal gene transfer from plants to bacteria. The first lateral transfer probably took place between plants and proteobacteria and the second one between plants and archaea after the duplication of the plant domain and the divergence between Hin1 and LEA^26^. This hypothesis is supported by the observation that the position of the *D*. *radiodurans* sequence in the Why domain tree does not reflect the usual phylogenetic relationships within bacteria^26^. *Deinococcus* bacteria are known to be prone to horizontal transfers^3, 6–10^. DrwH is a novel WHy domain-containing hydrophobic LEA5C protein exclusively present in the genus *Deinococcus*. This raises interesting questions concerning the evolution of putative LEA proteins within *Deinococcus* bacteria. Comparative genomic analysis showed that the gene cluster containing six genes (from *dr1369* to *dr1374*) in the *D*. *radiodurans* genome is interrupted by additional coding sequences in other *Deinococcus* strains, and is accompanied by gene rearrangement and gene truncation (see Supplementary Fig. S9). As expected, the gene subcluster containing the *dr1370*, *dr1371* and *drwH* genes displayed a high degree of similarity in all *Deinococcus* strains. This high conservation, combined with the rareness of the LEA genes in bacteria, suggests the possibility of acquisition by horizontal transfer. Furthermore, structural and functional properties of the WHy domains from DrwH and dWHy1 are schematically shown in Fig. 5, reflecting the divergent evolution due to different niches and selection pressures. In contrast to highly host-adapted pathogens and symbionts undergoing genome reduction, *Deinococcus* bacteria continually expand their genomic repertoires through many nearly simultaneous gene transfer events, leading to a highly adapted extremophile, which may contribute to their extreme tolerances. The results presented here provide new insight into the adaptive evolution and survival strategies of *Deinococcus* bacteria under extreme environmental conditions. Further investigation of the function and action of bacterial LEA proteins during abiotic stresses will be required to elucidate the molecular mechanisms underlying the extreme resistance of *D*. *radiodurans* in more detail.

## Materials and Methods

### Bacterial strains, plasmids and media

Strains and plasmids used in this study are described in Supplementary Table S2. *D*. *radiodurans* WT and its derivatives were cultured aerobically at 30 °C in TGY medium (1% tryptone, 0.5% yeast extract, and 0.1% glucose) in the presence of antibiotics as required. *E*. *coli* strains were grown in Luria-Bertani (LB) broth at 37 °C with appropriate antibiotics. Solid media contained 1.5% agar.

### qRT-PCR for gene expression

qRT-PCR was performed as previously described^46^. Primer pairs used for qRT-PCR are listed in Supplementary Table S3. The PCR reactions were carried out with an AB 7500 Real Time PCR System according to the manufacturer’s recommendations. See Supplementary Materials and Methods for more details.

### Construction of the *drwH* deletion mutant

The *drwH* deletion mutant was constructed using double crossover recombination of a kanamycin resistance cassette into the genome as described previously (see Supplementary Fig. S3)^34^. Primers were designed based on the full sequence of the *drwH* gene and are listed in Supplementary Table S3. Amplification of a 749-bp DNA fragment upstream of the *drwH* using the primer set P1/P2, a 1,007-bp kanamycin-resistance gene (*nptII*) from the plasmid pKatAPH3 using primes P3/P4, and a 594-bp DNA fragment downstream of the *drwH* using the set P5/P6 was performed. The three amplified products were used as templates for the overlap reaction as described previously^47^, and the resulting PCR fragment (2,268-bp) was directly transformed into *D*. *radiodurans* strain R1. Then, colonies resistant to kanamycin (20 μg/mL) were selected. The absence of the *drwH* gene in the mutant was verified by PCR and DNA sequencing (see Supplementary Fig. S3). When using the primer pair P7/P8, a band corresponding to the migration of a 346-bp molecule was detected in the WT strain; however, it was not detected when the analysis was performed with DNA extracted from the deletion mutant. In addition, PCR products with the primer pair P9/10 were sequenced; the results verified that the *nptII* gene was inserted into the right position and replaced the *drwH* gene. The resulting *drwH* deletion mutant, named R1-01 (Δ*drwH*), was used for further study.

### The construction of truncated Dr-WHy and dW-WHy proteins

The 306-bp DNA fragment of the *drwH* gene with the intact WHy domain but without the predicted signal peptide, whose product is designated Dr-WHy,  was amplified from genomic DNA of *D*. *radiodurans* using primers Dr-WHy-F (CGGGATCCATGCTCACGAGTTTCAGTTT) with a *BamH*I site and Dr-WHy-R (CCCAAGCTTTCACGTCAGAGTTCCGTCCA) with a *Hind*III site. Similarly, a 306-bp DNA fragmentof the *dwhy1* gene with the intact WHy domain but without the predicted signal peptide, whose product is designated dW-WHy, was amplified from a synthetic recombinant plasmid PUC57-*dwhy1* using the primers dW-WHy-F (CGGGATCCATGCTCGTTGATGTTGAACT) with a *BamH*I site and dW-WHy-R (CCCAAGCTTTTAAGTTTTTACCTCTGCAT) with a *Hind*III site. Two PCR products were digested with *BamH*I/*Hind*III and cloned into the pET28a vector. The resulting plasmids were transformed into the applicable *E*. *coli* host strain BL21 (DE3) to generate the truncated-DrwH strain BL21-1 and the truncated-dWHy1 strain BL21-2, respectively. The *E*. *coli* strain carrying the empty vector pET28a was designated BL21-0 as a control.

### Abiotic stress-resistance assays

The cells were treated with various concentrations of H_2_O_2_ and NaCl, different periods of desiccation, and two cycles of freezing and thawing as previously described^2, 28, 48^. Different dilutions of these cells were plated on agar plates and incubated at 30 °C (*D*. *radiodurans*) or 37 °C (*E*. *coli*) for 1-3 days before colonies were observed and enumerated. The survival rate was expressed as the percentage of the number of colonies in the treated samples compared with those in the untreated controls. All experiments were performed three times, and values are shown as the mean ± standard deviation. See Supplementary Materials and Methods for more details.

### Activity measurement of major antioxidant enzymes

The *D*. *radiodurans* WT and Δ*drwH* mutant cells (OD_600_≈0.6, treated with 50 mM H_2_O_2_ or not) were harvested by centrifugation, washed and resuspended with sterile PBS (pH 7.0), and disrupted on ice with an ultrasonicator. The debris was removed by centrifugation at 13,000 g at 4 °C for 20 min. Protein concentrations of the supernatants were determined by the Bradford method using BSA as the standard. The CAT activity was measured using spectrophotometric assays and calculated by monitoring the decrease in absorbance at 240 nm resulting from the disappearance of H_2_O_2_ as previously described^49^. The SOD activity was determined according to method of Beauchamp & Fridovich^50^, in which one unit of SOD was defined as the amount required to inhibit the photoreduction of nitroblue tetrazolium by 50%. The POD activity was measured according to method described by Omran^51^, in which one unit of POD was defined as the amount that oxidized 1 mol of diaminobenzidine tetrahydrochloride as a substrate. All experiments were repeated at least three times independently.

### MDH and LDH enzymatic activity measurement under oxidative stress *in vitro*

The protective effects of the purified intact recombinant Dr-WHy (truncated-DrwH) on MDH and LDH against oxidative stress were assayed as described previously^52, 53^ with some modifications. The enzymes and corresponding protein were diluted in 50 mM potassium phosphate buffer (pH 7.2) (MDH) or 25 mM Tris-HCl stabilizer solution (pH 7.5) (LDH) to a final concentration of 1.0 mg/mL following the manufacturer’s recommendations. Two concentrations of H_2_O_2_ (1.0 and 2.0 mM) were added to the enzyme mixture and incubated at room temperature for 1 hour. MDH and LDH activities were monitored as the rate of decrease in absorbance at 340 nm for 1 min due to the conversion of NADH into NAD^+^ at 25 °C. The rate determined for the untreated samples was considered to be 100% in all graphs. See extra details in Supplementary Materials and Methods.

## Electronic supplementary material

DrwH-Supplementary Information
