# Supplementary material for: DrwH, a novel WHy domain-containing hydrophobic LEA5C protein from Deinococcus radiodurans, protects enzymatic activity under oxidative stress
Source: Sci Rep. 2017 Aug 24;7:9281. doi: 10.1038/s41598-017-09541-2 (PMC5570939; doi:10.1038/s41598-017-09541-2)
Supplement: Supplementary file 1 — DrwH-Supplementary Information [file 41598_2017_9541_MOESM1_ESM.pdf]

Supplementary Information

DrwH, a novel WHy domain-containing hydrophobic LEA5C protein from *Deinococcus radiodurans*, protects enzymatic activity under oxidative stress

Shijie Jiang[1,2*], Jin Wang[2*], Xiaoli Liu[2], Yingying Liu[2], Cui Guo[2], Liwen Zhang[2], Jiahui Han[2], Xiaoli Wu[2], Dong Xue[2], Ahmed E. Gomaa[2], Shuai Feng[2], Heng Zhang[2], Yun Chen[2,3], Shuzhen Ping[2], Ming Chen[2], Wei Zhang[2], Liang Li[2], Zhengfu Zhou[2], Kaijing Zuo[3], Xufeng Li[1], Yi Yang[1]& Min Lin[2]

Supplementary Materials and Methods

Bioinformatics analysis. General protein characteristics, such as amino acid content, molecular weight and protein GRAVY, were predicted using the ProtParam tool (http://web.expasy.org/protparam/). A hydropathy plot was established with the ProtScale program (http://web.expasy.org/protscale/) using the Kyte and Doolittle algorithm[1]. SOPMA was used to predict the secondary structure of the protein[2] (https://npsa-prabi.ibcp.fr/cgi-bin/npsa_automat.pl?page=/NPSA/npsa_sopma.html) and regions of protein disorder were predicted using the Cspritz web server[3] (http://protein.bio.unipd.it/cspritz/) and the Gensilico Metadisorder service[4] (http://iimcb.genesilico.pl/metadisorder). Amino acid sequences harboring the Water stress and Hypersensitivity response domain were predicted from the SMART non-redundant database[5] (http://smart.embl-heidelberg.de/). Signal peptide prediction was performed using SignalP4.1[6] (http://www.cbs.dtu.dk/services/SignalP/) and PrediSi[7] (http://www.predisi.de/).

qRT-PCR for gene expression. Total cellular RNA was extracted from cultures at the beginning of the exponential phase using TRIzol Reagent (Invitrogen, Carlsbad, CA), and its quality and quantity were evaluated by UV absorbance at 260 and 280 nm. The RNA samples were reverse-transcribed using a Protoscript First Strand cDNA Synthesis Kit (New England Bio-Labs) as described in the manufacturer's protocol. The qRT-PCR assays were performed using total RNA samples obtained from three independent cultures. Optimized primers were designed using primer software and are listed in Table S3. The PCR reactions were carried out with an AB 7500 Real Time PCR System (Applied Biosystems, Foster City, CA, USA) according to the manufacturer's recommendations. The 16S rRNA gene was used as the endogenous reference control for normalization of differences in total RNA quantity, and relative gene expression was determined using the comparative threshold cycle $2^{-\Delta\Delta CT}$ method. All cDNA samples were assayed in triplicate[8].

Construction of the plasmid-mediated complementation strain of the *drwH* mutant. The plasmid carrying the wild-type *drwH* gene with its endogenous promoter was constructed using the *E. coli*-*D. radiodurans* shuttle plasmid pRADZ3 as a vector[9] (see Supplementary Fig. S8 online) to complement the constructed R1-01 deletion strain. Briefly, an 835-bp DNA fragment of the wild-type *drwH* gene with its endogenous promoter and terminator regions was amplified from genomic DNA of strain R1 with the primer P11 with a *Hind*III site and P12 with a *BamH*I site (see Supplementary Table S3 online). The amplicon was double-digested with *Hind*III and *BamH*I, and ligated into the corresponding site of pRADZ3 to yield the complementation plasmid pRA-*drwH*. Correct recombination was checked by PCR, followed by nucleotide sequencing of the amplicon obtained. pRA-*drwH* was transformed into R1-01 deletion strain to generate the plasmid-mediated complementation strain, designated R1-11 (Δ*drwHcomp*) (see Supplementary Table S2 online).

Abiotic stress-resistance assays. The *D. radiodurans* cells were grown in TGY medium with the appropriate antibiotics to the beginning of the exponential phase (OD_{600}≈0.6) at 30°C. Cultures were then pelleted from 1 mL cultures by centrifugation to remove the growth medium. For H_2O_2 treatment, the pelleted cells were treated with fresh TGY medium containing various concentrations (20, 40, 60, 80, 100 mM) of H_2O_2 in the dark for 30 min. For NaCl treatment, the prepared cells were resuspended in fresh TGY medium containing different concentrations (ranged from 0 to 5 M) of NaCl with shaking for 5 h. Desiccation stress assays were carried out as previously described[10] with some modifications. Briefly, 100 μL of cell suspension was placed inside a sealed desiccator at 25°C. Relative humidity within the desiccator was measured as less than 5% with a hygrometer. The desiccators were sealed, and the dried cultures were stored undisturbed at 25°C for 60 days. The samples were rehydrated by the addition of 1 mL TGY at regular intervals (ranged from 0 to 60 days) under sterilized conditions for 30 min. At the times indicated, 10 times serial dilutions were made, and 100 or 10 μL of each serial dilution of cell suspensions was spread/dripped onto TGY

agar plates. These plates were incubated at 30°C for 3 days before colony growth was observed and enumerated. The survival rate was expressed as the percentage of the number of colonies in the treated samples compared with those in the untreated controls. All the experiments are performed three times, and the values are shown as the mean ± standard deviation.

The wild-type and recombinant *E. coli* strains were grown in LB broth supplemented with kanamycin (50 μg/mL) and 0.1 mM isopropyl-thiogalactopyranoside (IPTG) at 37°C to an OD_{600} of 0.5. Cells were then pelleted from 1 mL cultures by centrifugation to remove the growth medium and resuspended in 1 mL fresh LB medium. For oxidative stress, 1.5 μL 30% H_2O_2 was added to a final concentration of 15 mM in the cell suspensions for 10 min. For freezing-thawing stress, 1 mL cell culture was frozen two times at -80°C for 20 min and thawed at room temperature for 20 min. After incubation, serial dilutions of 10 times were made. Ten microliters of each dilution was dripped onto LB agar plates at 37°C overnight. All assays were performed in triplicate.

Preparation of the Dr-WHy protein. The recombinant strain BL21-1 expressing Dr-WHy protein was grown in LB broth supplemented with kanamycin (50 μg/mL) at 37°C to an OD_{600} of 0.5 and then induced with 0.5 mM IPTG at 16°C overnight. Then cells were harvested and resuspended in 20 mM Tris-HCl, pH 8.0, and 300 mM NaCl. Cells were lysed by sonication on ice, and the lysates were cleared by centrifugation at 13,000 g for 20 min at 4°C. The supernatant was then subjected to affinity chromatography on Ni^{2+}-NTA agarose (Qiagen). After washing, the proteins were eluted with buffer (50 mM Tris-HCl, pH 8.0, 1 M NaCl) containing 300 mM imidazole and dialyzed overnight. The purity of purified Dr-WHy proteins was greater than 95%, as judged by electrophoresis analysis on a 15% polyacrylamide gel containing SDS that was then stained with Coomassie Blue (see Supplementary Fig. S6 online).

MDH and LDH enzymatic activities measurement under oxidative stress *in vitro*. MDH from porcine heart (Sigma) and LDH from rabbit muscle (Sigma) were used to test the protective effects of the Dr-WHy (truncated-DrwH) protein against oxidative stress according to a previously published method[11,12]. Experiments were performed in Eppendorf tubes to avoid protein adsorption to glass. The enzymes and corresponding protein were diluted in 50 mM potassium phosphate buffer (pH 7.2) (MDH) or 25 mM Tris-HCl stabilizer solution (pH 7.5) (LDH) to a final concentration of 1.0 mg/mL following the manufacturer's recommendations. To measure the protective effect of the Dr-WHy protein on MDH or LDH activity after oxidative treatment, Dr-WHy or BSA was added to equal volumes of MDH or LDH (10 μg/mL final concentration) at a molar ratio of 1:1 (test protein: enzyme). The stock solution of Tris-HCl buffer was used as a control. Two concentrations of H_2O_2 (1.0 and 2.0 mM) were added to the enzyme mixture and incubated at room temperature for 1 hour[12]. At the end of the treatment, the samples were transferred to wet ice until enzyme activity was determined. Assays were performed in triplicate, and the appropriate buffer solution without Dr-WHy protein was used as the blank. To determine MDH enzyme activity, 10 μL of LDH/stabilizer solution was added to 1 mL of 150 mM potassium phosphate buffer (pH 7.5) containing 200 μM oxaloacetate (Sigma) and 200 μM NADH (Sigma). For LDH, enzymatic activity was assayed in 100 mM KCl (pH 7.5) with 150 μM NADH (Sigma) and 2 mM pyruvate (Sigma). MDH and LDH activities were monitored as the rate of decrease in absorbance A_{340} nm for 1 min due to the conversion of NADH into NAD^+ at 25°C. Residual activity after treatment was expressed as percent of the initial activity. Enzyme activity for each sample shown was determined in at least three independent tests, as indicated in the figure legend for each experiment.

Supplementary Figures

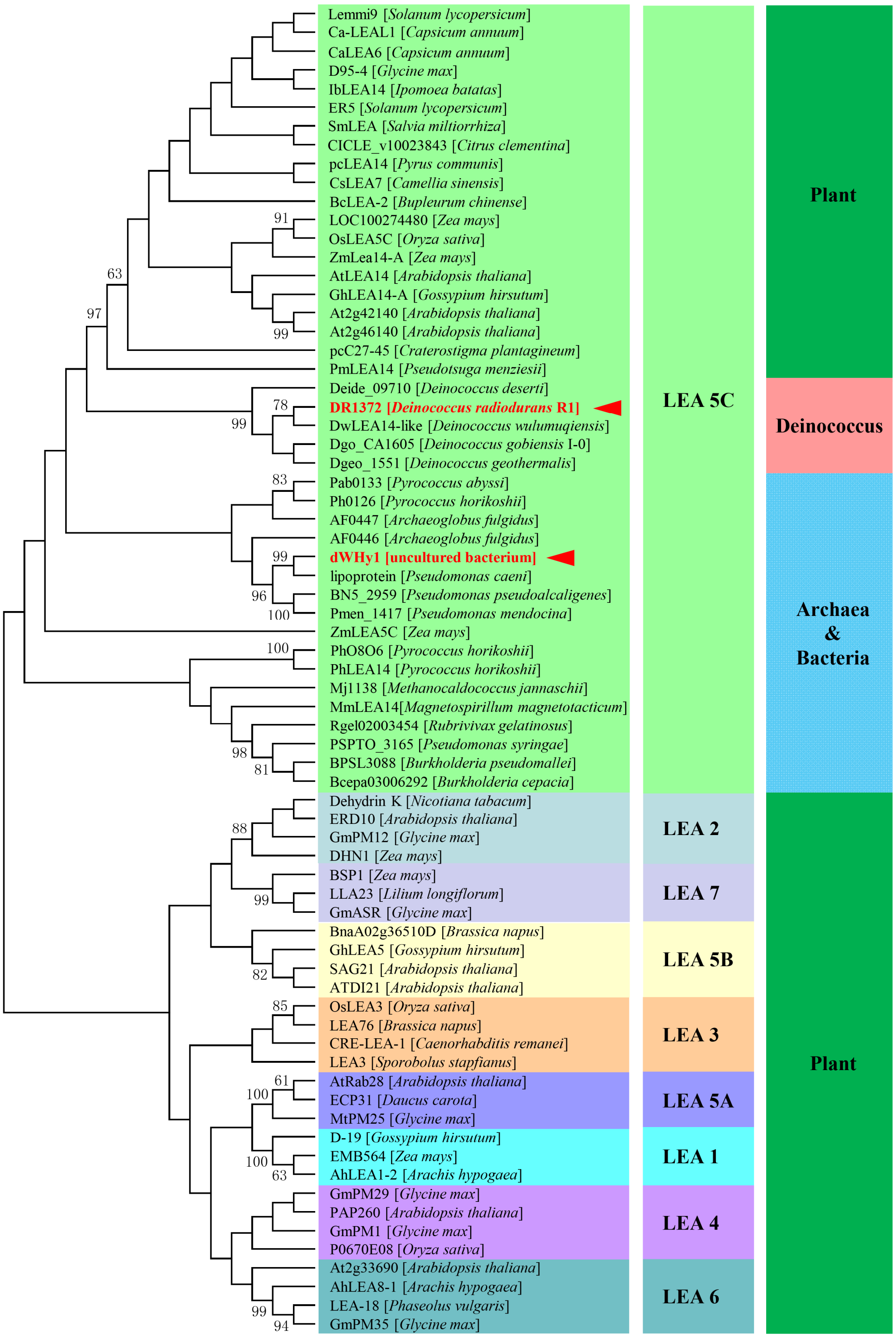

Figure S1. Phylogenetic tree of DrwH and related protein sequences.

Neighbor-joining (NJ) tree method was used to compute the distances of LEA5C proteins using the MEGA 7.0 program. The different LEA groups are shown by different colors. The *D. radiodurans* DrwH protein and the dWHy1 protein from uncultured bacteria are indicated with red arrows.

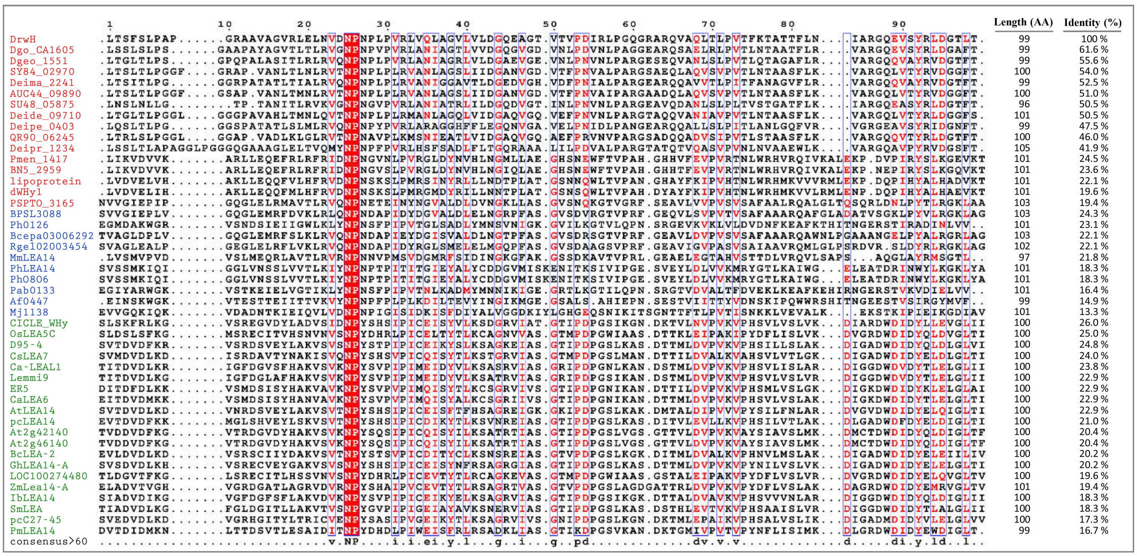

Figure S2. Multiple alignments of WHy domain from LEA5C proteins.

Alignment was performed using ClustalX2 and then manually refined. The bacterial sequences are labeled in red, archaea in blue, and plants in green. The consensus sequences with >60% identity were reported below the alignment. The lengths and identities of different WHy domains were calculated and listed.

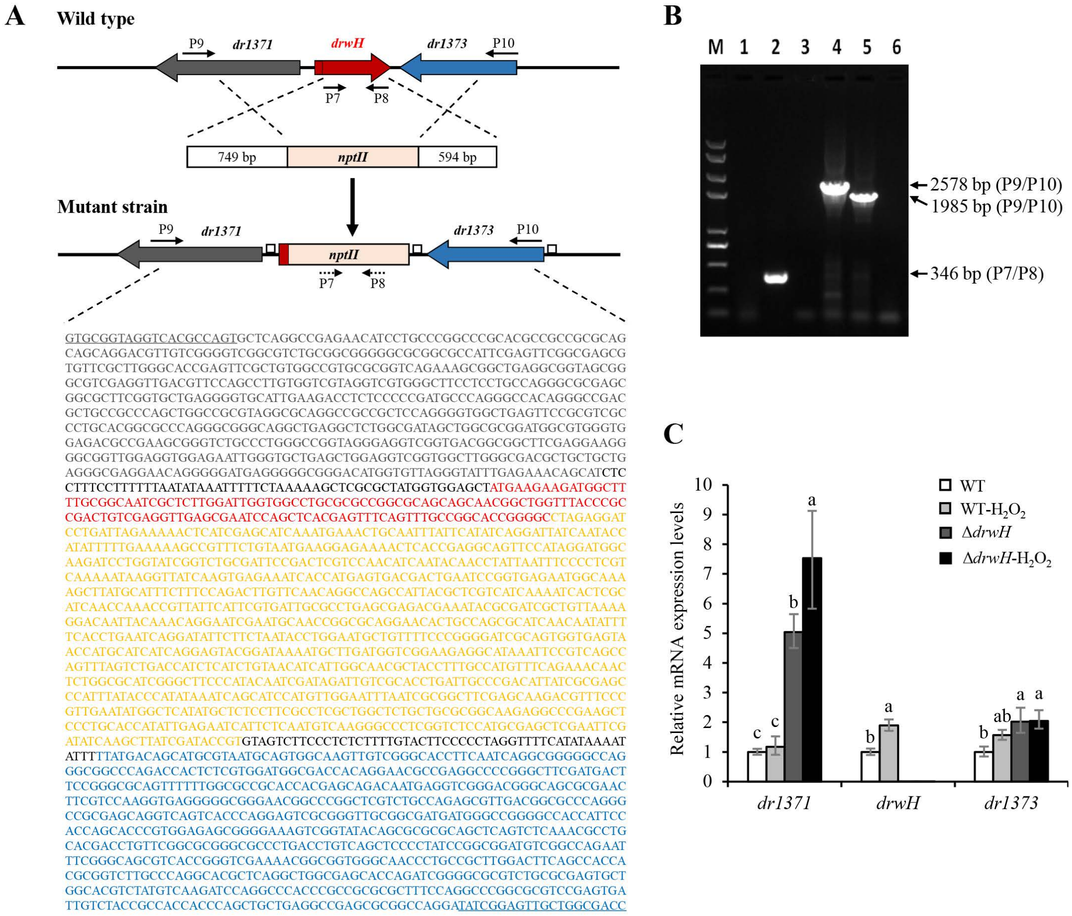

Figure S3. Construction and verification of the Δ*drwH* mutant.

(**A**) Schematic representation of the Δ*drwH* mutant generated by replacing the *drwH* region with the kanamycin resistance gene *nptII* (Kan^r). The open boxes represent the putative promoter regions of the flanking genes and *nptII* genes. (**B**) PCR verification of the Δ*drwH* mutant. Lane M: Trans2K PlusII DNA marker; lanes 1 & 4: PCR products amplified from the Δ*drwH* mutant using primers P7/P8 and P9/P10, respectively; lanes 2 & 5: PCR products amplified from *D. radiodurans* WT using primers P7/P8 and P9/P10, respectively; and lanes 3 & 6: PCR products amplified from sterile water using primers P7/P8 and P9/P10, respectively. (**C**) Effect of the *drwH* deletion on the expression of its flanking genes (*dr1371* and *dr1373*) under normal growth and oxidative stress conditions. Relative levels of transcripts are presented as the mean values ± standard deviation, calculated from three sets of independent experiments and normalized to levels in the WT strain. Different letters indicate significant differences (P< 0.05).

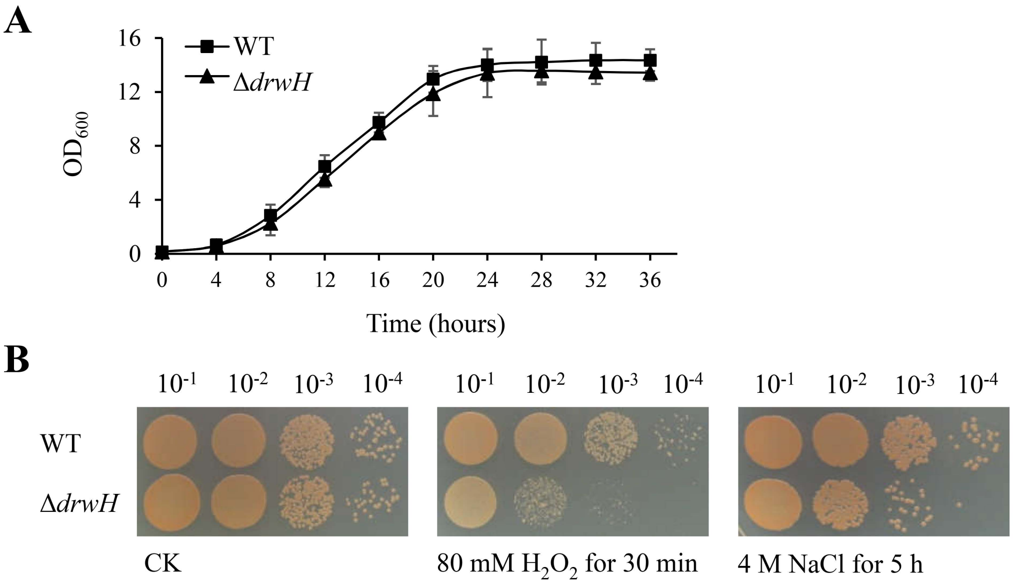

Figure S4. Survival phenotype plate assay upon oxidative or salinity stresses.

(**A**) Growth curves for *D. radiodurans* WT and Δ*drwH* mutant in TGY rich medium under normal conditions. All experiments are performed three times and values are mean ± standard deviation. (**B**) Serial 10-fold dilutions of OD-standardized *D. radiodurans* WT and Δ*drwH* mutant were spotted onto TGY plates after exposure to 80 mM H_2O_2 or 4 M NaCl. CK, untreated culture control. All experiments were performed three times.

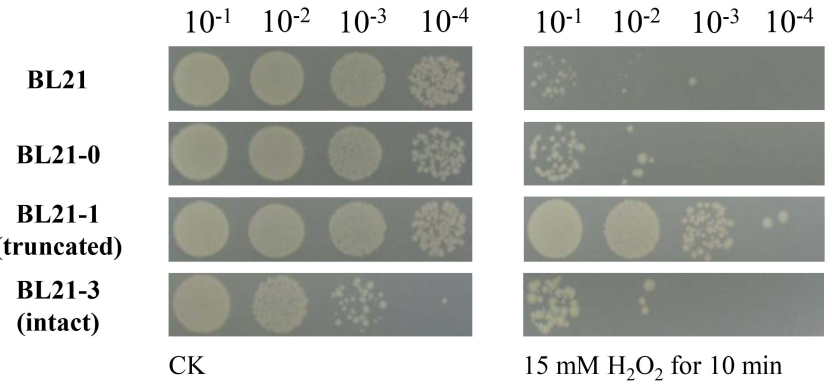

Figure S5. Survival phenotype plate assay upon oxidative stress.

Serial 10-fold dilutions of the OD-standardized wild-type *E. coli* BL21 and recombinant strains (the intact DrwH-expressing strain BL21-3, the truncated DrwH-expressing strain BL21-1, and the control strain BL21-0 harboring the empty vector pET28a) were spotted on LB plates after exposure to 15 mM H_2O_2. All experiments were performed three times.

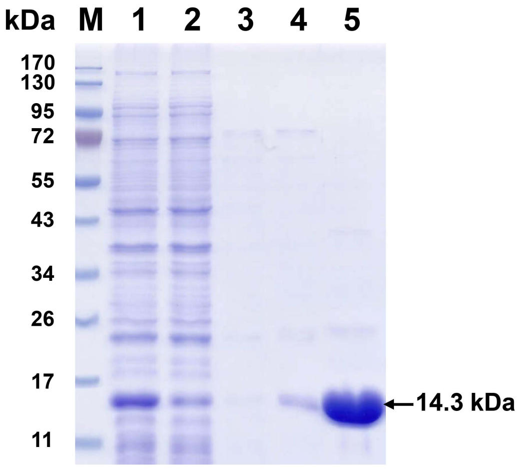

Figure S6. SDS-PAGE analysis of purified Dr-WHy.

Cultures were incubated with 0.5 mM IPTG at 16°C overnight. Lane M, molecular weight standards (kDa); lane 1, supernatant after lysis; lane 2, flow through; lane 3, elution with 10 mM imidazole; lane 4, elution with 50 mM imidazole; and lane 5, elution with 300 mM imidazole.

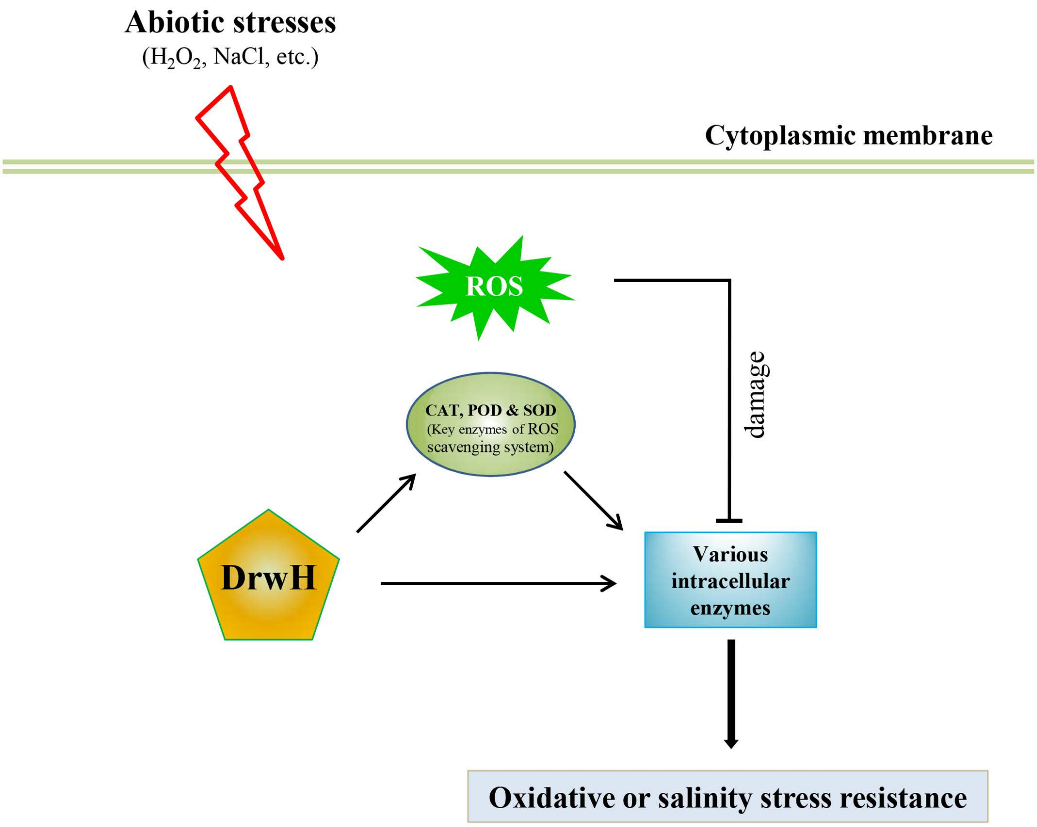

Figure S7. A proposed working model for DrwH in *D. radiodurans*.

The figure shows the proposed model in which DrwH effectively protect activities of various intracellular enzymes from damage caused by oxidative stress. Solid arrow and blocked arrow represent protection and damage effects, respectively. For more details, see the results or discussion of text.

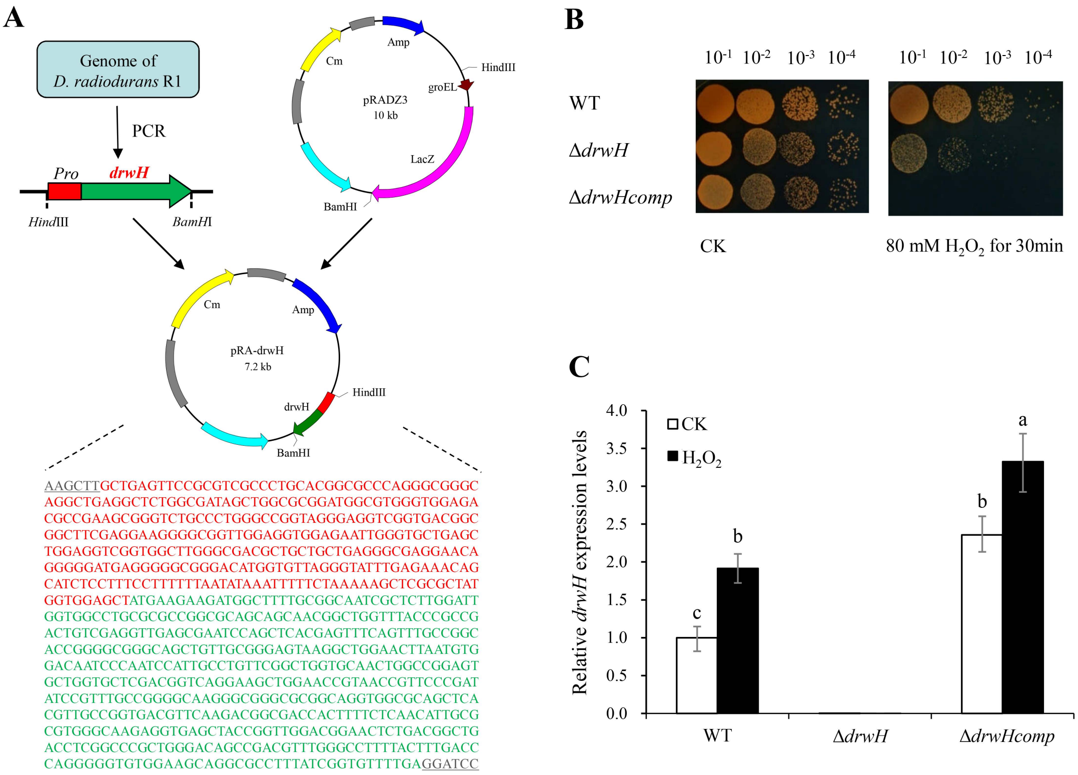

Figure S8. Complementation assays under oxidative stress.

(**A**) Construction and verification of the complementation plasmid pRA-*drwH*. (**B**) Survival phenotype plate assay upon oxidative stress. Serial 10-fold dilutions of OD standardized *D. radiodurans* WT, Δ*drwH* and Δ*drwHcomp* were spotted onto TGY plates after exposure to 80 mM H_2O_2 for 30 min. CK, untreated culture control. (**C**) *drwH* transcription in *D. radiodurans* WT, Δ*drwH* and Δ*drwHcomp* under 80 mM H_2O_2. Relative levels of transcripts are presented as the mean values ± standard deviation, calculated from three sets of independent experiments and normalized to levels in the WT strain. Different letters indicate significant differences ($P < 0.05$). All experiments were performed three times.

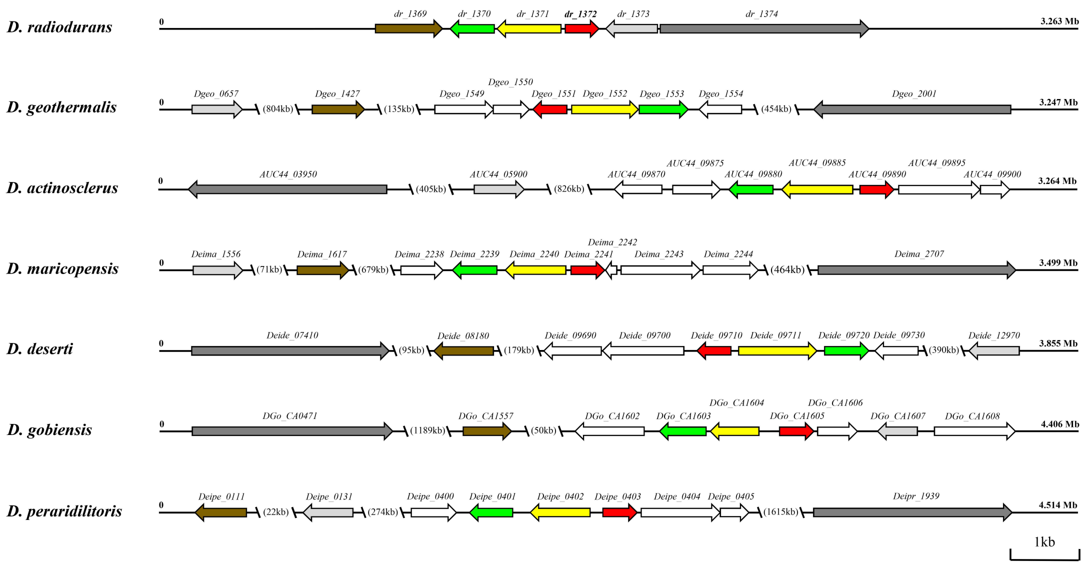

Figure S9. Physical organization and comparative analysis of the six gene clusters containing *drwH* of *D. radiodurans* compared with *D. geothermalis* DSM11300, *D. actinosclerus* BM2, *D. maricopensis* KR-23, *D. deserti* VCD115, *D. gobiensis* I-0, and *D. peraridilitoris* KR-200.

Same colors indicate similar predicted functions of the depicted ORF. The numbers in parentheses indicate the distances between the two genes on the left and right sides. Orthologous genes of *drwH* are shown in red. Orthologous genes of *dr1370* and *dr1371* are shown in yellow and green, respectively. Adjacent genes are labeled with other colors. The genome size is indicated at the end of physical organization.

Supplementary Tables

Table S1. Comparison of amino acid content (mol%), GRAVY and instability index of LEA

LEA group	Residue type<br>Residue groups	Small<br>Ala, Gly	Non-polar<br>Ile, Leu, Met, Val	Imino acid<br>Pro	Acid+amide<br>Asn, Asp, Gln, Glu	Basic<br>Arg, Lys, His	Hydroxyl<br>Ser, Thr	Hydrophilic<br>Acid+amide+ basic+hydroxyl	GRAVY	Instability index
5C	**DR1372**	**20.1**	**28.0**	**9.1**	**16.3**	**8.5**	**10.9**	**35.7**	**0.220**	**37.82**
	DGo_CA1605	23.4	29.4	10.4	21.0	7.3	10.4	38.7	0.366	45.22
	IbLEA14	16.9	27.5	6.9	19.9	11.2	9.3	40.4	0.031	6.70
	OsLEA5	11.9	28.5	7.3	16.6	11.3	15.2	43.0	0.020	23.54
	dWHy1	11.6	29.7	5.5	16.3	15.1	12.1	43.5	-0.087	27.79
	BN5_2959	12.5	27.2	6.6	20.6	18.3	6.6	45.5	-0.388	18.11
5A	MtPM25	28.5	20.2	4.7	22.5	9.8	11.8	44.1	-0.242	34.73
	ECP31	27.7	19.9	5.5	21.9	8.6	13.3	43.8	-0.275	39.82
	AtRab28	23.0	18.0	4.9	26.3	10.9	16.4	53.6	-0.564	37.20
5B	GhLEA5	21.0	18.0	4.0	13.0	14.0	18.0	45.0	-0.293	49.10
	SAG21	22.6	19.6	3.1	16.6	14.4	17.5	48.5	-0.360	41.83
	ATDI21	22.2	21.1	3.8	14.5	13.5	18.2	46.2	-0.254	43.73
1	D-19	23.5	13.7	2.0	29.3	16.6	11.8	57.7	-1.265	44.41
	AhLEA1-2	23.4	12.8	1.1	29.8	18.1	12.7	60.6	-1.377	45.85
	EMB564	25.3	12.1	1.1	24.2	18.7	16.5	59.4	-1.265	41.65
2	DHN1	23.6	12.1	3.2	19.1	19.7	17.8	56.7	-1.234	21.12
	Dehydrin K	17.4	10.2	8.2	28.6	24.5	7.1	60.2	-1.563	49.37
	ERD10	8.5	16.2	7.3	29.6	21.6	13.4	64.6	-1.348	58.15
3	OsLEA3	30.0	7.0	0.5	24.5	17.0	19.5	61.0	-1.065	25.10
	LEA76	21.1	8.3	1.1	27.5	15.0	20.7	63.2	-1.099	29.84
	CRE-LEA-1	26.6	6.8	0.4	27.9	19.4	13.0	60.3	-1.184	12.45
4	P0670E08	36.0	9.4	4.7	22.0	15.4	10.6	48.0	-0.821	34.14
	GmPM29	17.5	15.3	7.6	21.4	22.9	11.5	55.8	-1.102	48.27
	PAP260	19.4	14.8	9.0	18.6	23.9	9.7	52.2	-1.042	63.70
6	LEA-18	22.0	8.5	4.9	24.4	15.8	18.3	58.5	-1.267	43.06
	AhLEA8-1	18.1	10.7	7.4	28.8	15.0	16.0	59.8	-1.381	56.48
	GmPM35	21.1	11.7	6.3	26.4	12.7	17.9	57.0	-1.112	54.59
7	BSP1	28.9	8.1	1.5	20.7	23.7	4.5	48.9	-1.236	41.57
	GmASR	27.3	6.2	2.9	20.6	20.1	12.6	53.3	-1.303	37.27
	LLA23	19.0	11.2	1.4	24.6	25.4	9.8	59.8	-1.227	35.45

Note: Grey shading represents the hydrophobic LEA5 protein family.

Table S2. Bacterial strains and plasmids used in this study

Strains and plasmids	Genotype or description	Reference or source
**Bacterial Strains**		
***D. radiodurans* R1**		
WT	Wild-type, Chinese Culture Collection: CGMCC 1.633	Laboratory stock
R1-01 (Δ*drwH*)	R1 *drwH*-deletion mutant, Kan^r	This study
R1-11 (Δ*drwHcomp*)	R1-01 containing the complementation plasmid pRA-*drwH*, Kan^r and Cm^r	This study
R1-21 (Δ*irrE*)	R1 *irrE*-deletion mutant, Spe^r	Zhang *et al.*[13]
E. coli		
BL21	F^- *ompT* *hsdS*$_B$ (r_B^- m_B^-) *gal dcm* (DE3)	TransGen Biotech
BL21-0	The control strain harboring the empty vector pET28a, Kan^r	This study
BL21-1 (Dr-WHy)	The recombination BL21 strain containing pET-*Dr-WHy*, Kan^r	This study
BL21-2 (dW-WHy)	The recombination BL21 strain containing pET-*dW-WHy*, Kan^r	This study
BL21-3 (intact)	The recombination BL21 strain containing pET-*drwH*, Kan^r	Laboratory stock
**Plasmids**		
pET28a (+)	Kan^r *oripBR322 lacI^q T7p*	Novagen
pET-*drwH*	pET28a carrying the wild-type *drwH* gene under the control of T7 promoter, Kan^r	This study
pET-*Dr-WHy*	pET28a derivative plasmid carrying the truncated *Dr-WHy* gene with an intact WHy domain but without the predicted signal peptide, Kan^r	This study
pET-*dW-WHy*	pET28a derivative plasmid carrying the truncated *dW-WHy* gene with an intact WHy domain but without the predicted signal peptide, Kanr	This study
pRADZ3	The *E. coli*-*D. radiodurans* shuttle vector, Ap^r, Cm^r	Meima & Lidstrom[9]
pRA-*drwH*	pRADZ3 derivative carrying the wild-type *drwH* gene under the control of its endogenous promoter, Cm^r	This study

Table S3. List of primers used in this study

Gene/Fragment name	Primers	Sequence (5′–3′)	Amplicon size (bp)
drwH-U	P1	GAGTTCGGCGAGCGTGTT	749
	P2	GTTTTTCTAATCAGGATCCTCTAGGCCCCG GTGCCGGCAAACT	
nptII	P3	AGTTTGCCGGCACCGGGGCCTAGAGGATC CTGATTAGAAAAAC	1007
	P4	AAAGAGAGGGAAGACTACACGGTATCGAT AAGCTTGATAT	
drwH-D	P5	ATATCAAGCTTATCGATACCGTGTAGTCTTC CCTCTCTTT	594
	P6	GCGTGGTGGCTGAAGTCC	
YZdrwH	P7	TGCGGGAGTAAGGCTGGAACT	346
	P8	TCAAAACACCGATAAAGGCG	
YZdrwH-UKD	P9	GTGCGGTAGGTCACGCCAGT	2578
	P10	GGTCGCCAGCAACTCCGATA	
drwHcomp	P11	CCCAAGCTTGCTGAGTTCCGCGTCGC	835
	P12	CGGGATCCTCAAAACACCGATAAA	
drr06	RT16s-F	ATTCCTGGTGTAGCGGTG	146
	RT16s-R	CATCGTTTAGGGTGTGGAC	
dr1372 (drwH)	RT1372-F	AACCGTAACCGTTCCCGATAT	164
	RT1372-R	CGAGGTCAGCCGTCAGAGTT	
dr1998 (cat)	RT1998-F	ACCAACATCCAGTCGCAG	167
	RT1998-R	CCTTCACCCTGGTCGTTG	
drA0259 (cat)	RTA0259-F	TGGGCAAGATGGTCCTCG	113
	RTA0259-R	CAGCGGGTCGTTGGTGA	
dr1279 (sod)	RT1279-F	TGCCCTACGCTTACGACG	159
	RT1279-R	CGAGCTGCTGAATGAGTTGT	
dr1546 (sod)	RT1546-F	GCACCGCCACCTTTAGC	108
	RT1546-R	TGCAGTTCGGGTTCTCG	
drA0202 (sod)	RTA0202-F	AACCGCAGTCTGGTCATCC	174
	RTA0202-R	TTTCTTCGCGTCGTAGGC	
dr0644 (sod)	RT0644-F	CTGGCTCTGATCGCTCTGC	172
	RT0644-R	TGATGGTCGTGCGGGTC	
drA0145 (pod)	RTA0145-F	TTCCCGAGTCGTTCAAAGTG	133
	RTA0145-R	TCAGCGAAAATGGTCAGC	
drA0301 (pod)	RTA0301-F	CGCTTTCAGGAGGTGTTCG	114
	RTA0301-R	CGCCGAGTAGCGGTCATA	

Supplementary References

1. Kyte, J. & Doolittle, R. F. A simple method for displaying the hydropathic character of a protein. *J. Mol. Biol.* **157**, 105-132, doi:10.1016/0022-2836(82)90515-0 (1982).
2. Geourjon, C. & Deleage, G. SOPMA: significant improvements in protein secondary structure prediction by consensus prediction from multiple alignments. *Comput. Appl. Biosci.* **11**, 681-684, doi:10.1093/bioinformatics/11.6.681 (1995).
3. Walsh, I. *et al.* CSpritz: accurate prediction of protein disorder segments with annotation for homology, secondary structure and linear motifs. *Nucleic Acids Res.* **39**, W190-W196, doi:10.1093/nar/gkr411 (2011).
4. Kozlowski, L. P. & Bujnicki, J. M. MetaDisorder: a meta-server for the prediction of intrinsic disorder in proteins. *BMC bioinformatics* **13**, 111, doi:10.1186/1471-2105-13-111 (2012).
5. Letunic, I., Doerks, T. & Bork, P. SMART: recent updates, new developments and status in 2015. *Nucleic Acids Res.* **43**, D257-D260, doi:10.1093/nar/gku949 (2015).
6. Petersen, T. N., Brunak, S., von Heijne, G. & Nielsen, H. SignalP 4.0: discriminating signal peptides from transmembrane regions. *Nat. Methods* **8**, 785-786, doi:10.1038/nmeth.1701 (2011).
7. Hiller, K., Grote, A., Scheer, M., Münch, R. & Jahn, D. PrediSi: prediction of signal peptides and their cleavage positions. *Nucleic Acids Res.* **32**, W375-W379, doi:10.1093/nar/gkh378 (2004).
8. Zhan, Y. *et al*. The novel regulatory ncRNA, NfiS, optimizes nitrogen fixation via base pairing with the nitrogenase gene *nifK* mRNA in *Pseudomonas stutzeri* A1501. *P. Natl. Acad. Sci. USA* **201604514**, doi:10.1073/pnas.1604514113 (2016).
9. Meima, R. & Lidstrom, M. E. Characterization of the minimal replicon of a cryptic *Deinococcus radiodurans* SARK plasmid and development of versatile *Escherichia coli-D. radiodurans* shuttle vectors. *Appl. Environ. Microbiol.* **66**, 3856-3867, doi: 10.1128/AEM.66.9.3856-3867.2000 (2000).
10. Mattimore, V. & Battista, J. R. Radioresistance of *Deinococcus radiodurans*: functions necessary to survive ionizing radiation are also necessary to survive prolonged desiccation. *J. Bacteriol.* **178**, 633-637, doi:10.1128/jb.178.3.633-637.1996 (1996).
11. Reyes, J. L. *et al*. Hydrophilins from distant organisms can protect enzymatic activities from water limitation effects *in vitro*. *Plant Cell Environ.* **28**, 709-718, doi:10.1111/j.1365-3040.2005.01317.x (2005).
12. Liu, Y. *et al.* ZmLEA3, a multifunctional group 3 LEA protein from maize (*Zea mays* L.), is involved in biotic and abiotic stresses. *Plant Cell Physiol.* **54**, 944-959, doi:10.1093/pcp/pct047 (2013).
13. Zhang, C. *et al.* The Site-Directed A184S Mutation in the HTH Domain of the Global Regulator IrrE Enhances *Deinococcus radiodurans* R1 Tolerance to UV Radiation and MMC Shock. *J. Microbiol. Biotechnol.* **25**, 2125-2134, doi:10.4014/jmb.1507.07008 (2015).
